# Drugs and Medicines of North America

**Published:** 1887-02

**Authors:** 


Drugs and Medicines of North America. A Quarterly. Edited and published by J. U. and C. G. Lloyd, Cincinnati. 1886.

No. 2 of Volume II has come to hand.

Before placing it upon our files we must stop to notice a very complete article in it upon Asimina Triloba, or Papaw. Following the usual botanical description, with cuts of the several parts of the plant, the editors give its chemistry. They have isolated its alkaloid—Asiminine. A view of the

---

crystals under the microscope is added, and then follows a study of the physiological effects, by Professor Roberts Bartholow, of Jefferson Medical College, Philadelphia, and a statement of its homœopathic uses, by Dr. E. M. Hale, Chicago Homœopathic College.

According to the latter authority, a child that had been poisoned by eating the rind of the unripe fruit had violent fever, vomiting, and was covered with a bright scarlet eruption. Pulse was 130°, temperature 105°. Fauces were red and swollen, tonsils and submaxillary glands enlarged, diarrhœa of yellowish color. Each day pulse and temperature fell until they became normal, desquamation occurred, and a carbuncle formed on the thigh, continuing two weeks. The diarrhœa continued four weeks. The whole case strongly resembled an attack of scarlet fever. Four other children that had eaten the fruit in company with this child were attacked with similar symptoms, but in less degree. W. M. J.

